# QTc prolongation assessment in anticancer drug development: clinical and methodological issues

**DOI:** 10.3332/ecancer.2009.130

**Published:** 2009-01-12

**Authors:** G Curigliano, G Spitaleri, F de Braud, D Cardinale, C Cipolla, M Civelli, N Colombo, A Colombo, M Locatelli, A Goldhirsch

**Affiliations:** 1 Division of Medical Oncology, European Institute of Oncology, I.R.C.C.S., Milan, Italy; 2 New Drugs Development Unit, European Institute of Oncology, I.R.C.C.S., Milan, Italy; 3 Unit of Cardiology, European Institute of Oncology, I.R.C.C.S., Milan, Italy

## Abstract

Cardiac safety assessments are commonly employed in the clinical development of investigational oncology medications. In anti-cancer drug development there has been increasing consideration for the potential of a compound to cause adverse electrocardiographic changes, especially QT interval prolongation, which can be associated with risk of torsades de pointes and sudden death. Irrespective of overt clinical toxicities, QTc assessment can potentially influence decision making at many levels during the conduct of clinical studies, including eligibility for protocol therapy, dose delivery or discontinuation, and analyses of optimal dose for subsequent development. Given the potential for serious and irreversible morbidity from cardiac adverse events, it is understandable that cardiac safety results can have broad impact on study conduct and patient management. The methodologies for risk management of QTc prolongation for non cardiac drugs have been developed out of experiences primarily from drugs used to treat non life-threatening illnesses in a chronic setting such as antibiotics or antihistamines. Extrapolating these approaches to drugs for treating cancer over an acute period may not be appropriate. Few specific guidelines are available for risk management of cardiac safety in the development and use of oncology drugs. In this manuscript, clinical and methodological issues related to QTc prolongation assessment will be reviewed. Discussions about limitations in phase-I design and oncology drug development will be highlighted. Efforts are needed to refine strategies for risk management, avoiding unintended consequences that negatively affect patient access and clinical development of promising new cancer treatments. A thoughtful risk management plan generated by an organized collaboration between oncologists, cardiologists, and regulatory agencies to support a development programme essential for oncology agents with cardiac safety concerns.

## Introduction

Prolongation of the QT interval associated with the potential fatal arrhythmia known as ‘torsades de pointes’ (TdP) has been a common cause of withdrawal from the market for several drugs [1]. Clinical trial designs have been well described to identify and quantify QTc prolongation. In the absence of other completely reliable surrogate measure for the arrhythmogenic potential of a drug, the characterization of QT interval prolongation is now considered essential for most new drug-development programmes. The decision in oncology by a patient to receive, or by a physician to administer, a promising anti-cancer agent (or by a regulatory agency to approve one) is predicated on the assumption that the benefits of therapy outweigh the risks. Thus, although clinicians, members of regulatory bodies and drug developers may be able to predict that a given drug may carry some risks due to QTc prolongation, precise determination of relative risk vs benefit remains elusive for the development and application of many anti-cancer agents. In this review, we summarize recent experience in oncology regarding clinical predictors of drug-induced prolongation of the QT interval and torsades de pointes, consider how new molecular predictors of a drug’s activity might be incorporated into anti-cancer drug-development programmes and clinical practice, and suggest a general approach to drugs that are suspected of causing this problem.

## Historical background

TdP was first described in 1966 by the French cardiologist Dessertenne [2]. Until 1989, TdP was known to occur fairly frequently during the initiation of class III anti-arrhythmic drugs (so that patients were placed on telemetry during the initiation of such drugs), fairly rarely in patients receiving psychiatric drugs (so that nothing systematic was done for these patients) and occasionally in very sick hospitalized patients receiving intravenous erythromycin. In the fall of 1989, the first recognized case of QTc prolongation and TdP associated with terfenadine was described by physicians at the National Naval Medical Center in Bethesda, Maryland [3]. This was a landmark adverse drug reaction that triggered the awareness that many apparently benign non-cardiovascular drugs could occasionally have the unwanted ability to prolong cardiac repolarization and thus contribute to torsades de pointes in susceptible patients. In the process of elucidating the mechanism of terfenadine-associated QT prolongation and TdP, it was demonstrated that terfenadine, but not its active metabolite fexofenadine, had the unanticipated property of blocking IKr and thus prolonging the QT interval [4]. The first regulatory document addressing the evaluation of non-cardiovascular drugs to alter cardiac repolarization in humans was published in 1997 [5]. Subsequently, the Food and Drug Administration (FDA) and the International Conference on Harmonization (ICH) followed suit with the E14 guidance document entitled ‘The Clinical Evaluation of QT/QTc Interval Prolongation and Proarrhythmic Potential for Non-Antiarrhythmic Drugs’, that was accepted in 2005 [6]. In addition to traditional drug products, recent experiences have raised concerns about direct or indirect actions to prolong QTc and/or to induce arrhythmia by other agents, including biologics and hormones.

## Clinical Background

Multiple factors have been implicated in causing QT prolongation and TdP. Among these, improper use of QT-interval-prolonging medications deserves special attention [7,8]. To reduce the risk of torsades de pointes, clinicians from all therapeutic areas should understand the fundamentals of drug-induced QTc prolongation. Another issue is related to congenital syndromes involving QT-interval prolongation. A convergence of data obtained from clinicians, basic electrophysiologists, and geneticists have improved understanding of the mechanisms whereby drugs may cause this type of arrhythmia. Although it is convenient to think of QT prolongation as occurring because of either congenital or acquired abnormalities, the phenomenon may sometimes involve a gene–environment interaction. Pure congenital prolongation characterized by lifelong, ambient QT prolongation is rare but does carry a high risk of sudden death. Several forms of congenital LQTS have been reported, and three forms (LQT1, LQT2, and LQT3) have been well characterized in previous studies [9–11] (Table 1).

## What is the QT interval?

**Figure 1:**
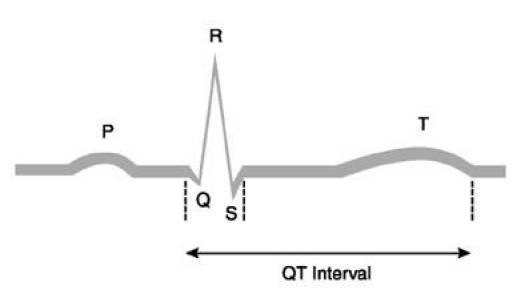
ECG representation and QT interval

The QT interval on the surface ECG is measured from the beginning of the QRS complex to the end of the T wave (Figure 1). It is the electrocardiographic manifestation of ventricular depolarization and repolarization. This electrical activity of the heart is mediated through channels, complex molecular structures within the myocardial cell membrane that regulate the flow of ions in and out of cardiac cells. The rapid inflow of positively charged ions (sodium and calcium) results in normal myocardial depolarization. When this inflow is exceeded by outflow of potassium ions, myocardial repolarization occurs. Malfunction of ion channels, which can result from drugs, electrolyte abnormalities, or other factors, leads to an intracellular excess of positively charged ions by way of an inadequate outflow of potassium ions or excess inflow of sodium ions. This intracellular excess of positively charged ions extends ventricular repolarization and results in QT-interval prolongation [12].

## Basic electrophysiological and genetic background

The ventricular action potential proceeds through five phases. The initial upstroke (phase 0—depolarization) occurs through the opening and closing of Na+ channels. The repolarization process begins with the rapid transient outflow of K+ ions (phase 1). This is followed by the flow of outward current through two delayed rectifier K+ channels (IKs, IKr) and of inward current through Ca2++ channels, constituting phase 2 or the plateau phase of repolarization. Increasing conductance of the rapid delayed rectifier (IKr) and inward rectifier (IK1) currents completes repolarization (phase 3). Phase 4 represents a return of the action potential to baseline. The LQT1 gene (also known as KCNQ1 and KvLQT1) encodes voltage-gated potassium channel alpha subunits. A tetramer of 4 KCNQ1 alpha subunits co-assembles with the minK gene product (beta regulatory subunit) to form the IKs slowly deactivating delayed rectifier potassium channel [13]. The patients with LQT1 account for about 42% of all patients with congenital LQTS [14,15]. The gene for LQT2 (also known as the KCNH2 and human ether-a-go-go-related or hERG gene) spans 55 kb and also encodes potassium channel alpha subunits. In terms of electrophysiology, hERG mutations cause potassium ion channels to deactivate (close) much faster, blunting the normal rise in current (IKr) that results from rapid recovery from channel inactivation/slow deactivation [13]. The LQT3 gene, SCN5A, encodes alpha subunits which form a fully sodium functional channel; beta subunits have a modifying influence. The gene responsible for LQT4, 220 kb in length, encodes the ankyrin-B (ANKB or ANK2) adaptor-protein. The LQT5 gene encodes the KCNE1 protein, which contains a single transmembrane spanning domain with small intra and extra-cellular components. The product of the minK gene forms the beta subunit of the LQT1 assembly regulating the IKs potassium channel current. The patients with this form of LQTS account for about 3% of all patients with LQTS. The LQT6 gene encoding MiRP1, or minK-related protein 1, is located 70 kb from minK on the same chromosome. The LQT7 genotype has been mapped to the inward rectifying potassium channel gene KCNJ2 on chromosome 17. The rare form of LQT7 (Andersen Syndrome) produces a combination of both a skeletal and cardiac muscle phenotype. The most recent addition, LQT8, has been mapped to the calcium channel gene CACNA1C on chromosome 12.

## Measurement and interpretation of the QT Interval

Because the QT interval is prolonged at slower heart rates and shortened at faster heart rates, many formulas have been proposed to adjust for these variations (Table 2). Yet differences of opinion exist regarding the most useful correction for heart rate [16–19]. One of the commonly used formulas is the Bazett formula, in which the QT interval is adjusted for heart rate by dividing it by the square root of the R–R interval. However, this formula has been criticized for being inaccurate at fast heart rates [20]. Other formulae are the Fridericia cube-root correction (QT interval divided by the cube root of the R–R interval) and the Framingham linear regression equation [17,18]. From an epidemiological perspective, the Framingham approach is supported because it is based on empirical data from a large population sample rather than on hypothetical reasoning. Analyses of QTc by the Fridericia correction (QTcF) has been considered for primary analysis (or co-primary along with Bazetts) in studies that are conducted in patients with malignancy, especially when increased heart rates are generally observed. Unfortunately, none of these corrections has been examined comparatively to determine the most effective formula in predicting which patients are at greatest risk of TdP.

## Factors that affect the QT interval

Although it is convenient to think of QT prolongation as occurring because of either congenital or acquired abnormalities, the phenomenon probably most often involves a gene–environment interaction. Pure congenital prolongation characterized by lifelong, ambient QT prolongation is rare but does carry a high risk of sudden death [21–27]. When exposed to QT-prolonging medications, individuals without lifelong QT prolongation may develop QT prolongation with or without TdP or may not develop QT prolongation at all. Even after adjustment for other factors that could prolong QT interval, some patients seem to be more likely than others to have QT prolongation at a given dose of a drug. This observation led researchers to hypothesize that patients with acquired QT prolongation may have a genetic predisposition for it. Recent investigations suggest that such patients may have clinically silent gene mutations that lead to overt QT prolongation only with exposure to QT-prolonging medications [12, 26, 27]. It is important to note that the majority of patients with documented acquired LQTS never experience TdP, and many patients with TdP have a normal QT interval shortly before the event. It appears that a variety of coincident circumstances, including genetic predisposition and a prolonged QT interval, are required to precipitate TdP. Factors that predispose to QT prolongation and higher risk of TdP include older age, female sex, low left ventricular ejection fraction, left ventricular hypertrophy, ischemia, slow heart rate, and electrolyte abnormalities including hypokalemia and hypomagnesemia [28–36]. A complete summary of factors affecting QT interval is reported in Table 3. Regarding antiarrhythmic QT-prolonging drugs, the risk of TdP seems to be highest within the first few days of starting therapy [37–40]. For this reason, physicians consider compulsive ECG monitoring at the initiation of treatment, including hospitalization that may be especially warranted in patients with established risk factors, since hospitalized patients can be better monitored for the warning signs that precede TdP. Tables 4 and 5, respectively, report drugs related to QTc prolongation and underlying molecular mechanisms.

## Correlation of QT prolongation with TdP or other clinically significant events

QT prolongation is a surrogate marker for the risk of developing TdP or other life-threatening arrhythmias. While there is a general qualitative correlation between QTc and risk for TdP, it is not possible to make a quantitative prediction for the risk of TdP for a given QTc prolongation. Thus while it makes sense not to accept a very small risk of TdP for a drug like terfenadine or cisapride, it is more difficult to know what degree of QTc prolongation would lead to such a large risk of TdP so as to make an anti-cancer drug unacceptable. One can measure the efficacy parameters in clinical trials, but regarding the risk for QTc, no study has made a quantitative assessment looking at any medication in the oncology setting. This is particularly difficult to assess because many cancer patients have many other factors that may increase their risk of arrhythmias including medication, cardiac disease, drug-induced heart injury and electrolyte abnormalities. The association of QT prolongation with arrhythmias can be influenced by a variety of factors mentioned in the previous section but can also be drug specific. For example, amiodarone can significantly prolong QTc, but it appears to cause only rare cases of TdP.

## QTc prolongation and anti-cancer drugs

The decision by a physician to use a drug is predicated on the assumption that the benefits of therapy, however defined, outweigh the risks, however measured. For a cancer patient, the possibility of a cure for the cancer may outweigh the potential risks of QTc prolongation, even when the prolongation is high.

### Arsenic trioxide

Arsenic trioxide (ATO) presents an interesting example of successful risk management, supporting the decision for a patient to accept, or a physician to administer an anti-cancer drug with established liabilities of QTc prolongation and TdP. Although this drug is known to provoke TdP [41] it is also uniquely effective in an otherwise fatal disease, relapsed acute promyelocytic leukaemia (APL) [42]. Therefore, until alternative therapy becomes available, arsenic trioxide remains a drug of choice, despite its potential for causing arrhythmia. In patients receiving multiple courses, QTc intervals may return to pre-treatment levels before the second course, signifying that serial ATO administration does not permanently prolong the QTc interval; however, documented episodes of TdP have been diagnosed beyond the first month of treatment, presumed due to drug accumulation in cardiac tissue [48]. Given appropriate ECG monitoring, identification of contributory factors, and management of electrolytes and concomitant medications, ATO can be safely administered [45, 48]. Two studies have demonstrated the efficacy of ATO in patients with relapsed acute promyelocytic leukaemia [43, 44]. In the first study patients experiencing first or two or more relapses were treated with daily ATO infusions to a maximum of 60 doses or until all leukemic cells in the bone marrow were eliminated. Thirty-four patients (85%) achieved a complete response. QTc prolongation was common (63%). The authors concluded that ATO provides important overall clinical benefit for patients with relapsed APL. However, cardiac toxicity occurs during ATO therapy, and patients should be monitored for prolonged QT intervals and ventricular arrhythmia [41,45–49]. Prolonged QT intervals developed in 38 patients (26 patients had intervals 500 milliseconds). All standard ECG tracings (25 mm/sec, 10 mm/m^2^) that had been obtained at baseline and during and following treatment with arsenic trioxide were collected and sent to a central core laboratory for review in a blind test by a single board-certified cardiac electrophysiologist. Baseline ECG abnormalities were observed in 36 patients, including marked sinus tachycardia, bundle branch block, ST-T wave changes, atrial fibrillation, and evidence of previous myocardial infarction. Despite these abnormalities, in 949 (80%) of the ECGs, the RR and QT intervals could be determined, and QTc intervals could be calculated. Compared with baseline, the heart rate-corrected (QTc) interval was prolonged by 30–60 msec in 36.6% of treatment courses, and by more than 60 msec in 35.4% of patients. The degree of prolongation was higher in men than in women during the first course of therapy, and in patients with hypokalemia. In patients receiving multiple courses, QTc intervals returned to pretreatment levels before the second course, signifying that arsenic trioxide does not permanently prolong the QTc interval [41]. In the early APL studies, the degree of QTc prolongation was higher in men than in women, and in patients with hypokalemia. One patient with relapsed APL with hypokalemia during arsenic trioxide treatment developed asymptomatic TdP, which resolved spontaneously and did not recur after electrolyte replacement [41, 45–47]. The risk for TdP has been associated with the magnitude of QTc prolongation, pre-existing QTc prolongation, a prior history of TdP, and congestive heart failure. One patient with APL developed this arrhythmia coincident with amphotericin administration, and the risk of TdP has been associated with concomitant QT-prolonging drugs. In addition to the potential for additive effects by drugs that are also known to prolong QTc, concomitant drugs can increase risk by other mechanisms, including: (a) drug–drug interaction that increases exposure of either agent to concentrations that have more significant effect on repolarization; and (b) electrolyte abnormalities. It has been demonstrated that ATO prolongs the action potential of guinea pig ventricular myocytes via two independent molecular mechanisms. ATO increases cardiac calcium currents, which regulate the plateau phase of the cardiac action potential but also reduces surface expression of the cardiac potassium current hERG/IKr, which is crucial to later stages of cardiac repolarization [50]. Enhanced outward currents and accelerated deactivation kinetics have been reported as a hallmark of hERG modulation by radical reactive oxygen species (ROS) [51,52] and are compatible with the well-documented property of ATO to induce oxidative stress by increasing ROS.

### Anthracycline based regimens

A prospective study [53] was designed to assess the effect of epirubicin-based chemotherapy on QT interval prolongation in patients with aggressive non-Hodgkin lymphoma, and potential impact of co-treatment by the cardioprotective agent dexrazoxane. Twenty untreated patients eligible for epirubicin-based chemotherapy were randomized to receive or not dexrazoxane hydrochloride after epirubicin infusion. Twelve-lead ECGs were recorded before and after epirubicin infusion and after dexrazoxane supplementation. All patients showed some QTc prolongation after chemotherapy infusion, but these effects were significantly reduced in the ten patients who also received dexrazoxane. The authors concluded that epirubicin-based chemotherapy causes an early increase of the QT and QTc prolongation, which is attenuated by dexrazoxane supplementation, and so dexrazoxane can hypothetically reduce the risk of arrhythmia in patients treated with epirubicin. In a previous prospective study, a Finnish group [54] investigated the effects of doxorubicin therapy on cardiac electrophysiology, with special emphasis on QT prolongation, in lymphoma patients who received doxorubicin to a cumulative dose of 400–500 mg/m^2^. Standard 12-lead ECG and signal-averaged ECG recordings were performed at baseline and after cumulative doxorubicin doses of 200, 400 and 500 mg/m^2^. Five patients (18%) developed QT prolongation exceeding 50 msec. The changes in QTc occurred independently of the impairment of left ventricular function. In children who received anthracyclines and thoracic irradiation, analysis of the 12-lead and 24-hour electrocardiograms demonstrated increased frequency of QTc prolongation, supraventricular premature complexes, supraventricular tachycardia, ventricular premature complexes, couplets and ventricular tachycardia when compared with an age-matched healthy population [55].

### Tamoxifen

Preclinical and clinical data demonstrated that tamoxifen can cause prolongation of the electrocardiographic QT interval in humans [56]. The electrophysiological mechanism(s) underlying the tamoxifen action are related to blocking the rectifier potassium current (IKr)

### Supportive care drugs

The anti-emetic efficacy of serotonin-type-3 (5-HT(3)) receptor antagonists has been found to be superior to older anti-emetic drugs in many clinical settings. Following the administration of these agents, changes in ECG parameters and increased or decreased heart rates have been demonstrated [57–59], but insufficient data are available that characterize the effects of newer anti-emetics on QTc in children or adults in the clinical treatment setting for advanced malignancy. The effect of droperidol on prolonging QT interval has been well described [59]. Some data suggest relatively modest QTc prolongation after administration of ondanestron or similar anti-emetics and lack of evidence for associated arrhythmias [57,59].However, methodological issues from published studies limit firm conclusions about the liability QTc from 5-HT(3) receptor antagonists, and the published results may not represent the effects expected from higher doses often used to manage chemotherapy-induced nausea and vomiting, so more clinical research is needed given the broad applications in many oncology treatment settings.

## QTc prolongation and anti-cancer drugs development

### Regulatory and methodological issues

New drugs present a special problem, because at the time of approval by the FDA or European Medicines Agency (EMEA), clinical experience with each drug is limited. Many new agents may weakly antagonize IKr or produce a small degree of QT-interval prolongation but there may be limited rigorous data at the high doses that are likely to be given to some patients post-approval. The decision to develop and approve an anti-cancer agent with QTc liability ultimately rests on an estimate of the perceived risk relative to the expected benefits for patients and society. Estimates of benefit are specific to particular indications, but they may include an assessment of whether the specific disease entity itself is associated with morbidity or mortality, the expected favourable impact of a new treatment, and consideration of efficacy and toxicity of other available therapies.

### Clinical trial evaluation ‘thorough QT/QTc study’

FDA and EMEA regulatory guidelines advocate that most new agents, administered systemically, should undergo an electrocardiographic evaluation, beginning early in clinical development, typically including a single trial dedicated to evaluating their effect on cardiac repolarization (‘thorough QT/QTc study’ or TQTS) [60]. Common conventions for conducting TQTS include digital data collection with rigorous methodology, appropriate rate corrections, categorical analysis (outlier), central tendency analysis and pharmacokinetic and pharmacodynamic analysis to enable analyses of exposure–effect relationships. The ability of a drug to prolong the QT/QTc interval is linked to pharmacologic effects that can be investigated in non-clinical models as well as clinically. At present, controversy exists about whether non-clinical testing can exclude a clinical risk of QT/QTc prolongation. TQTS would be needed in almost all cases when non-clinical data are not considered able to preclude the risk of QT/QTc prolongation. Additional factors that could influence the need for such a study include duration of treatment, metabolic profile, pharmacodynamic duration of action, and previous experience with other members of the same chemical or pharmacological class. At least two approaches can be described that correspond to different drug categories: (1) the TQTS for most drugs, and (2) the cardiology safety assessment often conducted as an ancillary sub-study in a phase-dose-escalation trial for cytotoxic or neuroleptic drugs (which could not be tested in healthy volunteers). The TQTS is a well-controlled double-blind study conducted in healthy volunteers. Commonly, a ‘crossover’ study design is employed where the ECG data are collected in the same patients before and during the treatment (drug-off and drug-on). For the drugs with a long half-life or with active metabolites and/or for schedules consisting of multiple doses, a ‘parallel group’ design can be employed. The positive control (whether pharmacological or non-pharmacological) should be well characterized and should consistently produce an effect corresponding to the largest change in the QT/QTc interval that is currently viewed as clinically not important. Studies should characterize the effect of a drug on the QT/QTc throughout the dosing interval. While the peak serum concentration does not always correspond to the peak effect on QT/QTc interval, care should be taken to perform ECG recordings at time points around the measured Cmax (maximal concentration). According to regulatory guidance about characterization of QTc prolongation in the early development of new therapeutic agents, until the effects of the drug on the QT/QTc interval have been characterized the following exclusion criteria are suggested: (1) a marked baseline prolongation of QT/QTc interval (e.g. repeated demonstration of a QTc interval >450); (2) a history of additional risk factors for TdP (e.g. heart failure, hypokalemia, family history of Long QT Syndrome); (3) the use of concomitant medications that prolong the QT/QTc interval. Regarding the development of oncology products, discussions continue between regulators and other stakeholders about the timing of QTc studies in early vs late development, and the applications of these exclusion criteria. The results of the TQTS will influence the amount of information collected in later stages of development: (a) a negative TQTS, even in the presence of non-clinical data of concern, will almost always allow the collection of on-therapy ECGs in accordance with the current practices in each therapeutic area to constitute sufficient evaluation during subsequent stages of drug development; (b) a positive TQTS will almost always call for an expanded ECG safety evaluation during later stages of drug development. For development of anti-cancer and other special agents, clinical protocol designs to characterize QTc prolongation remains an area of active research and discussion among regulators and the pharmaceutical industry. The TQTS design is not readily applicable to cytotoxics or other oncologics, especially the agents that cannot be administered to healthy volunteers at therapeutic or super-therapeutic exposures. Cardiac safety is often evaluated in phase-I studies, conducted in patients with advanced malignancy. Clearly, oncology phase-I studies are not commonly randomized (crossover or parallel) with placebo groups, and they often lack the internal controls and eligibility requirements used in the TQTS. There are no specific rules in the E14 (ICH guidelines) which apply for special drugs such as those in oncology. E14 specifically recognizes that the ‘thorough study’ cannot be performed with cytotoxic drugs in healthy volunteers. The E14 only states ‘other ways of detecting effects on the QT/QTc interval need to be developed. These might include the collection of ECGs at multiple time points under tightly controlled settings that target a broad range of doses early in development’.

## QTc prolongation in oncology drug development plans

When one is prepared to take a drug into clinical development for a specific oncology indication, a clinical development plan must consider the degree of risk for QT prolongation. If the risk is small, based on known class effects and preclinical information, then the detailed evaluation of QTc risk can be deferred to the phase-II programme (or phase III), and the phase-I programme can just have routine surveillance ECGs. If the drug has a significant risk of QT prolongation prior to clinical trials, then it is important to evaluate that risk in the phase-I trials. Whatever the plan, it should have feedback and approval in advance from the regulatory agencies at the appropriate times. The degree of QT prolongation that is acceptable when making a decision if a drug should go into development or continue development is highly dependent on commercial, medical and regulatory variables that are not easy to integrate. While more than a 5 msec increase is not acceptable for some drugs, the arsenic trioxide example demonstrates that a drug with a much longer increase can have approval. The clinical development plan must prospectively take into consideration what degree of prolongation would be acceptable, and undertake trials that can document the degree of QT prolongation that the drug actually causes. In other words, the trials must provide the investigators and sponsor the information to continue or stop development, to provide the regulatory agencies the information they need to approve or disapprove the drug, and ultimately provide the physicians the information they need to proceed. All of these decisions need specific considerations in oncology.

## Clinical and methodological considerations in phase-I oncology trials

Several levels of concern can be highlighted in designing phase-I trials in oncology, that may be different from other indications (patient concerns, protocol design issues, and regulatory issues). First of all are patient concerns: (1) the safety of the patient is of paramount importance. Based on drug pharmacokinetics and known preclinical or clinical signals for risk of QTc prolongation, an appropriate safety plan for monitoring QTc and responding to potential changes must be put in place. This can range from periodic ECG data collection and reading, that can have a turnaround measured in days for oral medications with low risk, to rapid QTc assessments based on bedside machine readouts for immediate decisions as to possible IV-drug discontinuation for medication with high risk. The medical team should have sufficient experience and training for an optimal safety assessment and subsequent decision-making; (2) patient capabilities and compliance in tolerating trial demands must be considered—the timing of the ECG is usually concomitant to pharmacokinetic blood sampling and requires outstanding compliance from the patient enrolled into the study; (3) for drugs with a significant risk of QTc prolongation, rapid assessment and decision making during administration of the drug can induce psychological distress that needs to be mitigated through education and preparation including the informed consent process.

Another level of concern is related to protocol issues. Trial protocols described in E14 have many features feasible in healthy volunteers or in patients from many therapeutic areas, but are not consistent with conventional care of patients with advanced malignancy enrolled in oncology trials. Oncology trial design issues can be divided into trial design issues and technical ECG issues. The trial design issues come from defining the purpose of the study and how the potential QT evaluation will affect the trial. For example, if there is no known QT prolongation risk for the drug, then routine surveillance ECGs can be done at baseline, at peak drug concentrations, and at a delayed time in case the QT prolongation is related to a metabolite of tissue accumulation. Another example would be to establish QT prolongation only for what might be life-threatening levels (>500 msec) without trying to document small changes. The definition of dose-limiting toxicities (DLTs) is important in phase I trials. The CTCAEv3 definition of Grade 3 QT prolongation is defined as QTc >500 msec without life threatening arrhythmias. Definition of clinical trial inclusion criteria is another important topic. In two studies, the appropriate reference values for adult patients with solid tumours, enrolled across various phase-I programmes were evaluated [61,62]. Data indicated that the distribution of QTc values increased when compared with results from a trial with similar ECG methods conducted in healthy volunteers. This implies that if exclusion criteria of 450 msec were considered, more than 10% of patients from phase-I or phase-II studies would be excluded because of marginally prolonged QTc at baseline. In view of the major difficulties in conducting a formal TQTS for an oncology product, it is not conceivable to exclude all patients with prolonged QTc at baseline. Such exclusions are often problematic for patients and caregivers, given the perceptions that phase-I studies offer access to new agents that can provide some opportunity for disease control; that the risk of QTc prolongation implies rare or poorly defined relationship to significant arrhythmia or other adverse clinical outcome; and of the relative risks of alternative treatments or disease morbidities.

Many technical protocol decisions related to the ECGs need to be made. These issues can be approached essentially in the same way in oncology trials as in trials in any other area, except that the special needs of oncology patients must be recognized. E14 addresses these technical issues in general ways, and more details are provided in other publications [63]. Measurement of QTc is highly variable. It is well described that measurements taken minutes apart typically differ by up to 40 msec, variations that do not correlate with known physiological differences. Thus important decisions such as eligibility, DLTs, drug interruptions, should not be made on single measurements but one should average multiple measurements taken minutes apart.

## Conclusion

Clinicians are increasingly faced with both old and new anti-cancer agents that have the potential to prolong the QT interval; a laboratory finding that is associated with the risk of significant arrhythmia. As more sensitive methods for electrocardiographic testing are applied, an increasing number of anti-cancer agents or treatments for supportive care will likely be described with some risk for QTc prolongation. For many agents, the frequency of QTc prolongation may be common while the risk of clinical arrhythmia (TdP) may be very rare. To enable the development and application of future treatments, detailed analyses and publication about TdP events, observed in cancer treatment settings, should continue to support rational strategies for risk management with broad relevance. Oncologists have demonstrated their ability to identify and manage complicated cardiac risks in clinical investigations and general practice. Across multiple geographic regions, successful risk management is exemplified by the published experiences with arsenic trioxide, highlighting the value of systematic ECG testing and attention to concomitant medications, electrolyte abnormalities, and co-morbidities, all likely contributing to the low frequency of clinical cardiac toxicities reported post-approval. Given the growing introduction of promising agents, designed to address unmet medical needs, efforts are needed to promote strategies for risk management, avoiding unintended consequences that can impede development, regulatory approval, and patient access. More research is needed to assess and manage cardiovascular safety of patients treated with anticancer agents, building from organized collaborations between oncologists and cardiologists, and these efforts should have broad relevance to therapeutics designed for treatment or supportive care.

Risk–benefit assessments might be relatively straightforward in non-oncological studies and are provided in the ICH E14 document. In principle, risk–benefit must be interpreted in the context of the nature and severity of the disease and conservative approaches will delay or prevent patient access to innovative treatment. Currently, different criteria are used in defining which prolongation of the QT-QTc time or change over baseline is feasible. The use of uniform thresholds to describe changes of concern for all protocol applications can simplify study conduct and subsequent data collection. Grading according to the Common Toxicities Adverse Events version 3 (CTCAE v3) might be considered a uniform guideline in which grade 3 QT-QTc prolongation is defined as a value exceeding 500 msec. In oncology trials, treatment related events of grade 3 or higher are often considered for decision making and dosing decisions. Asymptomatic prolongation of the QT-QTc time exceeding 60 msec over baseline may be too sensitive to guide dosing in oncological studies, since these changes can already be observed as a diurnal variation, even in healthy volunteers, and might even be larger in oncological patients. Of utmost importance is that the data generated in oncological studies are pooled to provide additional information on the QT/QTc interval in cancer patients and to guide us in broadening the eligibility criteria of studies in the oncologic setting, to implement the concomitant use of oncology relevant medication, to standardize dose-modification and discontinuation criteria, to search for alternatives in study design and result in timely approval of drugs by the regulatory authorities providing access for patients to promising new anti-cancer agents.

## Search strategy and selection criteria

Material included in this review was identified from computerized searches of PubMed (1966 to September 2008), EMBASE (1974 to September 2008) and Current Contents (1998 to September 2008) using the medical subject headings (MeSH) terms ‘QTc prolongation or QT interval or arrhythmia, anticancer drugs. Free text terms ‘QTc prolongation* AND chemotherapy AND phase I’ were also used and combined with ‘clinical trials’, ‘epidemiologic studies’, ‘animal studies’, and ‘in-vitro studies’. Only peer-reviewed English-language papers were eligible for inclusion. Additional information and studies were obtained by checking the reference lists from retrieved papers. EMEA and FDA guidelines for QTc prolongation assessment were also considered in this review.

## Figures and Tables

**Table 1:**
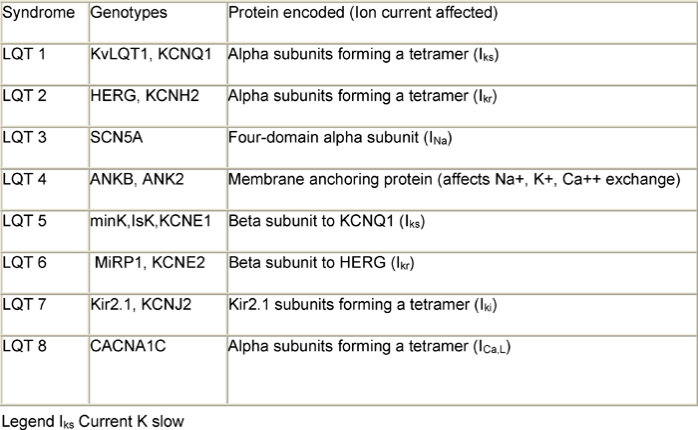
LQT syndromes

**Table 2:**
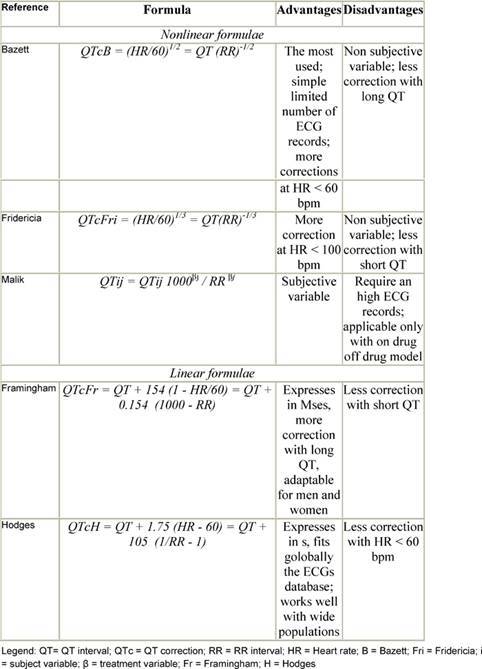
QT correction formulae

**Table 3:**
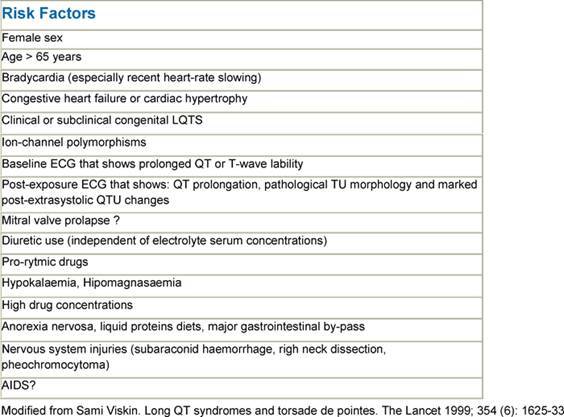
Risk Factors for torsade de pointes

**Table 4:**
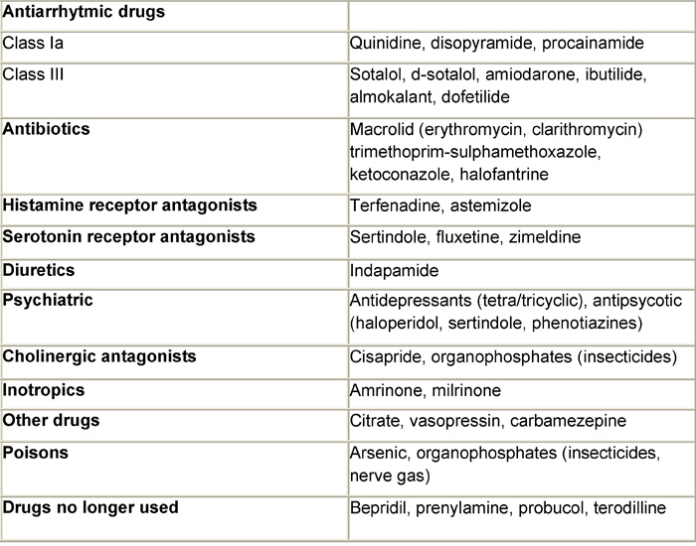
Drug inducing QTc prolongation

**Table 5:**
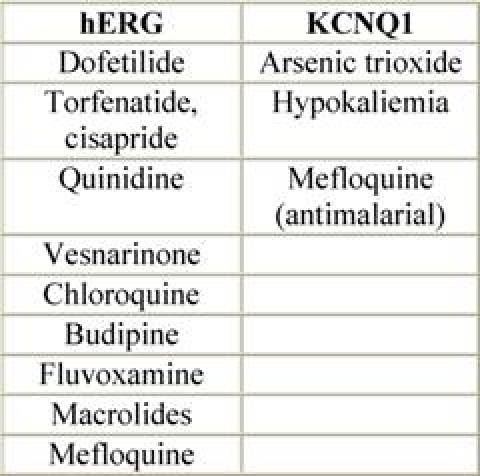
Ion channels and QT prolonging drugs

## References

[1] Roden DM (2004). Drug-Induced Prolongation of the QT Interval. N Engl J Med.

[2] Dessertenne F (1966). La tachycardie ventricularire a deux foyers opposes variables. Arch Mal Coeur.

[3] Monahan BP, Ferguson CL, Killeavy ES, Lloyd BK, Troy J, Cantilena LR (1990). Torsades de pointes occurring in association with terfenadine use. JAMA.

[4] Pratt C, Brown AM, Rampe D, Mason J, Russell T, Reynolds R, Ahlbrandt R (1999). Cardiovascular safety of fexofenadine HCl. Clin Exp Allergy.

[5] The Clinical Evaluation of QT/QTc Interval Prolongation and Proarrhythmic Potential for Non-antiarrhythmic Drugs. www.fda.gov/ohrms/dockets/ac/03/briefing/pubs%5Cprelim.pdf.

[6] Food and Drug Administration (2005). International Conference on Harmonisation; guidance on E14 Clinical Evaluation of QT/QTc Interval Prolongation and Proarrhythmic Potential for Non-Antiarrhythmic Drugs. Fed Regist.

[7] (1999). MedWatch **Glaxo Wellcome voluntarily withdraws Raxar (grepafloxacin)** [press release].

[8] FDA Talk Paper (2000). Janssen Pharmaceutica Stops Marketing Cisapride in the US. http://www.fda.gov/ohrms/dockets/ac/00/backgrd/3634b1a_tab4a.htm.

[9] Chiang CE, Roden DM (2000). The long QT syndromes:genetic basis and clinical implications. J Am Coll Cardiol.

[10] Viskin S (1999). Long QT syndromes and torsade de pointes. Lancet.

[11] Moss AJ, Zareba W, Benhorin J (1995). ECG T-wave patterns in genetically distinct forms of the hereditary long QT syndrome. Circulation.

[12] Morganroth J, Brozovich FV, McDonald JT, Jacobs RA (1991). Variability of the QT measurement in healthy men, with implications for selection of an abnormal QT value to predict drug toxicity and proarrhythmia. Am J Cardiol.

[13] Vincent GM (2000). Long QT syndrome. Cardiol Clin.

[14] Splawski I, Shen J, Timothy KW, Lehmann MH (2000). Spectrum of mutations in long-QT syndrome genes: KVLQT1, HERG, SCN5A, KCNE1, and KCNE2. Circulation.

[15] Shimizu W, Antzelevitch C (2000). Differential effects of betaadreneric agonists and antagonists in LQT1, LQT2, LQT3 models of the long QT syndrome. J Am Coll Cardiol.

[16] Funck-Brentano C, Jaillon P (1993). Rate-corrected QT interval: techniques and limitations. Am J Cardiol.

[17] Hnatkova K, Malik M (1999). ‘Optimum’ formulae for heart rate correction of the QT interval. Pacing Clin Electrophysiol.

[18] Aytemir K, Maarouf N, Gallagher MM, Yap YG, Waktare JE, Malik M (1999). Comparison of formulae for heart rate correction of QT interval in exercise electrocardiograms. Pacing Clin Electrophysiol.

[19] Malik M (2001). Problems of heart rate correction in assessment of drug-induced QT interval prolongation. J Cardiovasc Electrophysiol.

[20] Milne JR, Ward DE, Spurrell RA, Camm AJ (1982). The ventricular paced QT interval: the effects of rate and exercise. Pacing Clin Electrophysiol.

[21] Schwartz PJ, Priori SG, Spazzolini C (2001). Genotype-phenotype correlation in the long-QT syndrome: gene-specific triggers for life-threatening arrhythmias. Circulation.

[22] Wilde AA, Jongbloed RJ, Doevendans PA (1999). Auditory stimuli as a trigger for arrhythmic events differentiate HERG-related (LQTS2) patients from KVLQT1-related patients (LQTS1). J Am Coll Cardiol.

[23] Schwartz PJ, Priori SG, Locati EH (1995). Long QT syndrome patients with mutations of the SCN5A and HERG genes have differential responses to Na+ channel blockade and to increases in heart rate: implications for gene-specific therapy. Circulation.

[24] Zhang L, Timothy KW, Vincent GM (2000). Spectrum of ST-T-wave patterns and repolarization parameters in congenital long-QT syndrome: ECG findings identify genotypes. Circulation.

[25] Moss AJ, Zareba W, Kaufman ES (2002). Increased risk of arrhythmic events in long QT syndrome with mutations in the pore region of the human ether-a-go-go-related gene potassium channel. Circulation.

[26] Priori SG, Napolitano C, Schwartz PJ (1999). Low penetrance in the long-QT syndrome: clinical impact. Circulation.

[27] Donger C, Denjoy I, Berthet M (1997). KVLQT1 C-terminal missense mutation causes a forme fruste long-QT syndrome. Circulation.

[28] Moss AJ, Schwartz PJ, Crampton RS (1991). The long QT syndrome: prospective longitudinal study of 328 families. Circulation.

[29] Reardon M, Malik M (1996). QT interval change with age in an overtly healthy older population. Clin Cardiol.

[30] Ahnve S (1985). QT interval prolongation in acute myocardial infarction. Eur Heart J.

[31] Rebeiz AG, Al-Khatib SM (2001). A case of severe ischemia-induced QT prolongation. Clin Cardiol.

[32] Khan IA (2002). Clinical and therapeutic aspects of congenital and acquired long QT syndrome. Am J Med.

[33] Kay GN, Plumb VJ, Arciniegas JG, Henthorn RW, Waldo AL (1983). Torsade de pointes: the long-short initiating sequence and other clinical features: observations in 32 patients. J Am Coll Cardiol.

[34] Roden DM (1994). Risks and benefits of antiarrhythmic therapy. N Engl J Med.

[35] Roden DM (1998). Mechanisms and management of proarrhythmia. Am J Cardiol.

[36] Makkar RR, Fromm BS, Steinmen RT, Meissner MD, Lehmann MH (1993). Female gender as a risk factor for torsades de pointes associated with cardiovascular drugs. JAMA.

[37] Maisel WH, Kuntz KM, Reimold SC (1997). Risk of initiating antiarrhythmic drug therapy for atrial fibrillation in patients admitted to a university hospital. Ann Intern Med.

[38] Chung MK, Schweikert RA, Wilkoff BL (1998). Is hospital admission for initiation of antiarrhythmic therapy with sotalol for atrial arrhythmias required? yield of in-hospital monitoring and prediction of risk for significant arrhythmia complications. J Am Coll Cardiol.

[39] Torp-Pedersen C, Moller M, Bloch-Thomsen PE (1999). Dofetilide in patients with congestive heart failure and left ventricular dysfunction. N Engl J Med.

[40] Simons GR, Eisenstein EL, Shaw LJ, Mark DB, Pritchett EL (1997). Cost effectiveness of inpatient initiation of antiarrhythmic therapy for supraventricular tachycardias. Am J Cardiol.

[41] Ohnishi K, Yoshida H, Shigeno K, Nakamura S, Fujisawa S (2001). Prolongation of the QT interval and ventricular tachycardia in patients treated with arsenic trioxide for acute promyelocytic leukemia. Ann Intern Med.

[42] Huan SY, Yang CH, Chen YC (1985). Arsenic trioxide therapy for relapsed acute promyelocytic leukemia: an useful salvage therapy. Leuk Lymphoma.

[43] Soignet SL, Frankel SR, Douer D, Tallman MS, Kantarjian H, Calleja E (2001). United States multicenter study of arsenic trioxide in relapsed acute promyelocytic leukemia. J Clin Oncol.

[44] Camacho LH, Soignet SL, Chanel S, Ho R, Heller G, Scheinberg DA (2000). Leukocytosis and the retinoic acid syndrome in patients with acute promyelocytic leukemia treated with arsenic trioxide. J Clin Oncol.

[45] Barbey JT, Soignet S (2001). Prolongation of the QT interval and ventricular tachycardia in patients treated with arsenic trioxide for acute promyelocytic leukemia. Ann Intern Med.

[46] Barbey JT, Pezzullo JC, Soignet SL (2003). Effect of arsenic trioxide on QT interval in patients with advanced malignancies. J Clin Oncol.

[47] Unnikrishnan D, Dutcher JP, Garl S, Varshneya N, Lucariello R, Wiernik PH (2004). Cardiac monitoring of patients receiving arsenic trioxide therapy. Br J Haematol.

[48] Unnikrishnan D, Dutcher JP, Varshneya N, Lucariello R (2001). Torsades de pointes in 3 patients with leukemia treated with arsenic trioxide. Blood.

[49] Naito K, Kobayashi M, Sahara N, Shigeno K, Nakamura S, Shinjo K, Tobita T, Takeshita A, Ohno R, Ohnishi K (2006). Two cases of acute promyelocytic leukemia complicated by torsade de pointes during arsenic trioxide therapy. Int J Hematol.

[50] Ficker E, Kuryshev YA, Dennis AT, Obejero-Paz C (2004). Mechanisms of Arsenic-Induced Prolongation of Cardiac Repolarization. Mol Pharmacol.

[51] Taglialatela M, Castaldo P, Iossa S, Pannaccione A, Fresi A, Ficker E, Annunziato L (1997). Regulation of the human ether-a-go go related gene (HERG) K+ channels by reactive oxygen species. Proc Natl Acad Sci USA.

[52] Berube J, Caouette D, Daleau P (2001). Hydrogen peroxide modifies the kinetics of HERG channel expressed in a mammalian cell line. J Pharmacol Exp Ther.

[53] Galetta F, Franzoni F, Cervetti G, Cecconi N, Carpi A, Petrini M, Santoro G (2005). Effect of epirubicin-based chemotherapy and dexrazoxane supplementation on QT dispersion in non-Hodgkin lymphoma patients. Biomed Pharmacother.

[54] Nousiainen T, Vanninen E, Rantala A, Jantunen E, Hartikainen J (1999). QT dispersion and late potentials during doxorubicin therapy for non-Hodgkin’s lymphoma. J Intern Med.

[55] Larsen RL, Jakacki RI, Vetter VL, Meadows AT, Silber JH, Barber G (1992). Electrocardiographic changes and arrhythmias after cancer therapy in children and young adults. Am J Cardiol.

[56] Liu XK, Katchman A, Ebert SN, Woosley RL (1998). The antiestrogen tamoxifen blocks the delayed rectifier potassium current, IKr, in rabbit ventricular myocytes. J Pharmacol Exp Ther.

[57] Pinarli FG, Elli M, Dagdemir A, Baysal K, Acar S (2006). Electrocardiographic findings after 5-HT(3) receptor antagonists and chemotherapy in children with cancer. Pediatr Blood Cancer.

[58] Buyukavci M, Olgun H, Ceviz N (2005). The effects of ondansetron and granisetron on electrocardiography in children receiving chemotherapy for acute leukemia. Am J Clin Oncol.

[59] Charbit B, Albaladejo P, Funck-Brentano C, Legrand M, Samain E, Marty J (2005). Prolongation of QTc interval after postoperative nausea and vomiting treatment by droperidol or ondansetron. Anesthesiology.

[60] The clinical evaluation of QT/QTc interval prolongation and pro-arrhythmic potential for non-antiarrhythmic drugs.

[61] Piekarz RL, Frye AR, Wright JJ (2006). Cardiac studies in patients treated with depsipeptide, FK228, in a phase II trial for T-cell lymphoma. Clin Can Res.

[62] Varterasian M, Meyer M, Fingert H, Radlowski D, Asbury P, Zhou X, Healey D (2003). Baseline heart rate-corrected QT and eligibility for clinical trials in oncology. J Clin Oncol.

[63] Morganroth J, Gussak I (2005). Cardiac Safety of Noncardiac Drugs: Practical Guidelines for Clinical Research and Drug Development.

